# Health Care Professionals’ Clinical Perspectives on Glycemic Control and Satisfaction With a New Blood Glucose Meter With a Color Range Indicator: Online Evaluation in India, Russia, China, and the United States

**DOI:** 10.2196/diabetes.9143

**Published:** 2018-01-09

**Authors:** Mike Grady, Laurence Barry Katz, Pamela Anderson, Brian Leonard Levy

**Affiliations:** 1 LifeScan Scotland Ltd Inverness United Kingdom; 2 LifeScan Inc West Chester, PA United States; 3 Johnson & Johnson Health Care Systems Inc Titusville, NJ United States

**Keywords:** color range indicator, blood glucose meter, self-monitoring of blood glucose, health care professionals

## Abstract

**Background:**

We previously demonstrated in patients with diabetes that displaying blood glucose results in association with color improved their ability to interpret glucose results.

**Objective:**

The objective of this study was to investigate the perceptions of health care professionals (HCPs) in specific countries about the value of color on a new glucose meter and to determine if HCP perspectives among countries differ on the value of this approach in clinical practice.

**Methods:**

A total of 180 HCPs, including 105 endocrinologists, 34 primary care physicians, 25 diabetes educators, and 16 pharmacists, were recruited from India (n=50), Russia (n=50), China (n=50), and the United States (n=30). These HCPs experienced the OneTouch Select Plus Simple glucose meter online from their own office computer using interactive demonstrations (webpages, meter simulator, and video clips). After providing demographic and current clinical practice insights, HCPs responded to questions about the utility of the color-enhanced glucose meter.

**Results:**

Mean age and years in their current professional role for the 180 HCPs was 41.3 (SD 8.1) and 13.3 (SD 6.8) years for endocrinologists, 41.3 (SD 8.3) and 14.1 (SD 6.8) years for primary care physicians, 37.5 (SD 8.7) and 12.7 (SD 6.8) years for diabetes educators, and 35.9 (SD 5.3) and 9.5 (SD 5.2) years for pharmacists. In all, 88% (44/50) of Russian and 83% (25/30) of American HCPs said their patients find it easy to recognize low, in-range, or high blood glucose results compared to 56% (28/50) of HCPs in China and 42% (21/50) in India. Regardless of country, HCPs had less confidence that their patients act on blood glucose results with 52% (26/50) in Russia, 63% (19/30) in the United States, 60% (30/50) in China, and 40% (20/50) in India responding positively. During the interactive online meter experience, HCPs from all countries responded positively to questions about a meter with color features. After reflecting on the value of this meter, most HCPs strongly agreed or agreed their patients would be more inclined to act on results using a meter with color features (Russia: 92%, 46/50; United States: 70%, 21/30; China: 98%, 49/50; India: 94%, 47/50). They also said that color was particularly useful for patients with lower numeracy or education who may struggle with interpreting results (Russia: 98%, 49/50; United States: 77%, 23/30; China: 100%, 50/50; India: 82%, 41/50).

**Conclusions:**

This multicountry online study provides evidence that HCPs had high overall satisfaction with the OneTouch Select Plus glucose meter, which uses color-coded information to assist patients with interpreting blood glucose results. This may be especially helpful in patient populations with low numeracy or literacy and limited access to health care and direct interaction with HCPs.

## Introduction

Guidelines suggest that when prescribing self-monitoring of blood glucose, health care professionals (HCPs) should ensure patients with diabetes receive ongoing instruction on interpreting blood glucose data so they may make lifestyle or therapy changes [1]. However, evidence from clinical practice in many countries, including China, Russia, and India, suggests patients struggle to achieve glycemic targets. A study in China found that 55% of 2819 insulin-treated patients with type 2 diabetes (T2D) had a glycated hemoglobin A_1c_ (HbA_1c_) greater than 8%, with 59% of patients reporting that they only occasionally follow their HCP’s instructions regarding self-monitoring of blood glucose [2]. A pharmacoepidemiological study observed a similar pattern of poor glycemic control in patients with T2D from 45 different towns in Russia reporting that 36% of patients had an HbА_1с_ greater than 8% [3]. Furthermore, a mobile diabetes project in rural Russia in patients with T2D found that access to HCPs and ongoing support for patients is problematic in these areas [4]. Lack of consistent contact with HCPs and limited understanding of self-monitoring of blood glucose can have a negative effect on maintaining positive self-care behaviors in these countries. For example, in one rural area of India only 25% of patients had performed even a single blood glucose test in the time between face-to-face doctor visits, a finding partly attributed to a lack of knowledge about how to perform the test [5]. In addition, even for patients who regularly attended a tertiary care hospital in India, self-care practices were found to be unsatisfactory and the authors recommended that more effort be directed toward educating people with diabetes in India [6].

Appropriate education addressing how to interpret self-monitoring of blood glucose information and how to respond to “out-of-range” results have been identified as important requirements for useful self-monitoring of blood glucose practice [7]. However, lack of the ability to interpret or act on self-monitoring of blood glucose can be compounded by other factors. For example, disparities in literacy, presence of literacy but lack of health literacy, and low numeracy across patients in various countries can impede efforts to support patients who struggle to comprehend self-care guidance or use the self-monitoring technologies provided by HCPs. For example, low diabetes-related numeracy skills are associated with fewer self-management behaviors [8] and poor numeracy is also associated with suboptimal glycemic control in patients with T2D [9] and type 1 diabetes (T1D) [10]. In addition to issues with numeracy, a recent UNESCO report found only 29% of countries are expected to achieve universal adult literacy targets with the number of illiterate adults worldwide projected to be 743,000,000 by 2015 [11]. Therefore, providing patients with glucose monitoring tools that are easy for HCPs to teach and easy for patients to interpret is important, especially in countries where both low numeracy and literacy are barriers to diabetes self-management. We previously reported that glucose meters utilizing color range indicators (ColorSure technology) improved the ability of patients with T1D and T2D to interpret glucose results [12]. In this study, we solicited feedback from HCPs in China, India, and Russia regarding a glucose meter that has features targeted to areas with diverse patient populations facing challenges in terms of access to health care (eg, in rural areas) or barriers to self-management (eg, lower literacy or numeracy). For comparison purposes, we also surveyed a cohort of HCPs providing diabetes care within the US health care environment.

## Methods

### Materials

**Figure 1:**
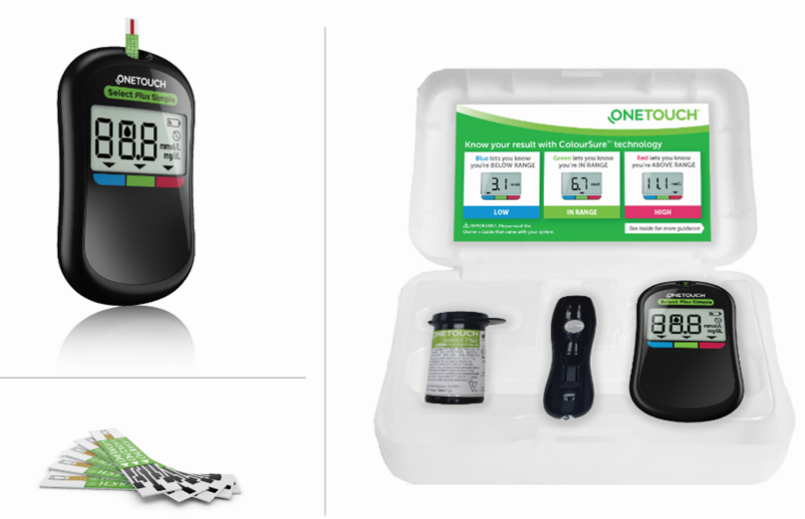
OneTouch Select Plus Simple blood glucose monitoring system components. An arrow pointing to the color bar on the meter casing indicates if the current blood glucose result is low (blue bar), in range (green bar), or high (red bar) to a target blood glucose range. The system uses OneTouch Select Plus blood glucose test strips and Delica lancing devices and contains a reference information card in the system kit that has space for health care professionals to write instructions and advice to their patients.

The OneTouch Select Plus Simple meter (LifeScan, Wayne, PA, USA) is intended for self-testing by people with diabetes as an aid to monitor the effectiveness of diabetes control. It is simple to use, has a small and slim design, no buttons to push, and a large visual display with big, easy-to-read numbers. The meter automatically lets patients know if their blood glucose result is below, above, or within a target glucose range by displaying the current blood glucose result with a range indicator arrow (ColorSure technology) pointing to a corresponding color bar below the meter display (blue for low; green for in range; red for high) (Figure 1). The meter also emits a fast audible beep when the blood glucose result is low and a slow audible beep when the blood glucose result is high for an added level of safety. The system comes with a paper-based reference card guide that the doctor, diabetes educator, or other HCP can fill out with individualized reminders of when to perform glucose tests and how a patient should respond to certain blood glucose results.

### Procedure

**Figure 2:**
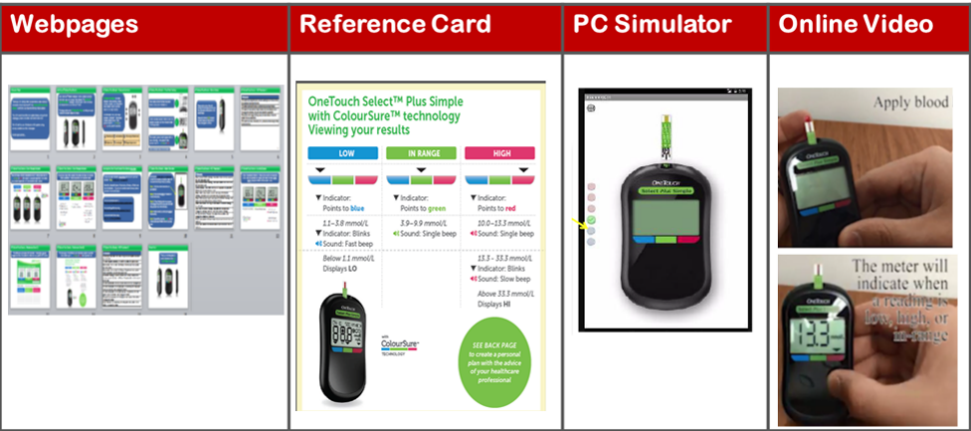
Methodology for the online health care professional (HCP) study. The HCPs interacted with webpages online describing features of the OneTouch Select Plus Simple, a reference card that was contained in the meter kit, an interactive simulation of use of the meter, and an online video demonstrating the proper use and features of the meter.

This multicountry online survey study was conducted by individual HCPs from institutions and clinical practices within each country. Webpages were provided to the HCP that summarized the features and benefits of the meter. In addition, short video clips pertaining to the setup and test process when using the meter were provided. An interactive computer simulation of the actual meter was provided online to allow each HCP to control and experience the various key features of the meter (Figure 2). A total of 180 HCPs from four countries (50 each from Russia, India, and China, and 30 from the United States) were recruited and included endocrinologists, primary care physicians, diabetes educators, and pharmacists.

Before the online experience with the meter, all HCPs provided demographic and clinical practice metrics with respect to the number and types of patients they routinely advised or treated.

The HCPs were then asked four clinical practice questions to determine the confidence they had in the ability of their patients to interpret or act on blood glucose results and to determine how often they provided insight on these topics to their patients. Participating HCPs then used the interactive online tool to experience the identical capability, functionality, and navigation as the intended product. The meter simulator was preloaded with representative low, in-range, and high blood glucose results or information that provided examples of the meter screens that appeared whenever HCPs (or patients) reviewed information. The HCPs interacted online with a series of 19 webpages displaying both text and visuals of the meter, with embedded links at various points which automatically gave the HCP a hands-on interaction (via mouse) with the meter simulator (Figure 2). In addition, participants viewed two videos showing real-time meter setup and routine glucose testing with the meter. At various stages during these activities, 25 survey questions were presented to assess the HCP’s opinions of the value of various functions and features of the meter to them and their patients. After completing the meter experience activities, the HCPs were asked three clinical practice-based questions pertaining to the value of the meter in supporting their patients with diabetes self-management and whether the meter might have particular benefits for patients with low numeracy.

### Statistical Analyses

Continuous demographic variables were described as median and range or mean and standard deviation. Categorical demographic variables were described as percentages within categories and are presented with both numerator and denominators. Patient responses to survey statements were recorded using a five-point Likert scale with a favorable response (4 or 5) deemed statistically significant if the lower 95% one-sided confidence limit for the percentage of participants providing a favorable response per item was greater than 50%.

## Results

### Health Care Professionals’ Demographic and Clinical Practice Information

A total of 180 HCPs took part in the study with 50 HCPs each in Russia, India, and China, and 30 HCPs from the United States. Professional background of the HCPs included 105 endocrinologists, 34 primary care physicians, 25 diabetes educators, and 16 pharmacists (Table 1). Pharmacists were not recruited as part of the US cohort of HCPs. Mean age across all four countries was mean 41 (SD 8) years (endocrinologists), mean 41 (SD 8) years (primary care physicians), mean 37 (SD 9) years (diabetes educators), and mean 36 (SD 5) years (pharmacists). Mean time in current role was mean 13 (SD 7) years (endocrinologists), mean 14 (SD 7) years (primary care physicians), mean 13 (SD 7) years (diabetes educators), and mean 10 (SD 5) years (pharmacists). The proportions of patients with T1D and T2D, respectively, typically seen by each professional in routine clinical practice was 20% and 80% (endocrinologists), 18% and 82% (primary care physicians), 32% and 69% (diabetes educators), and 23% and 77% (pharmacists). Country-specific variations in HCP demographics and clinical practice parameters are shown in Table 1.

### Health Care Professionals’ Current Clinical Practice Feedback on Patient Self-Care

**Figure 3:**
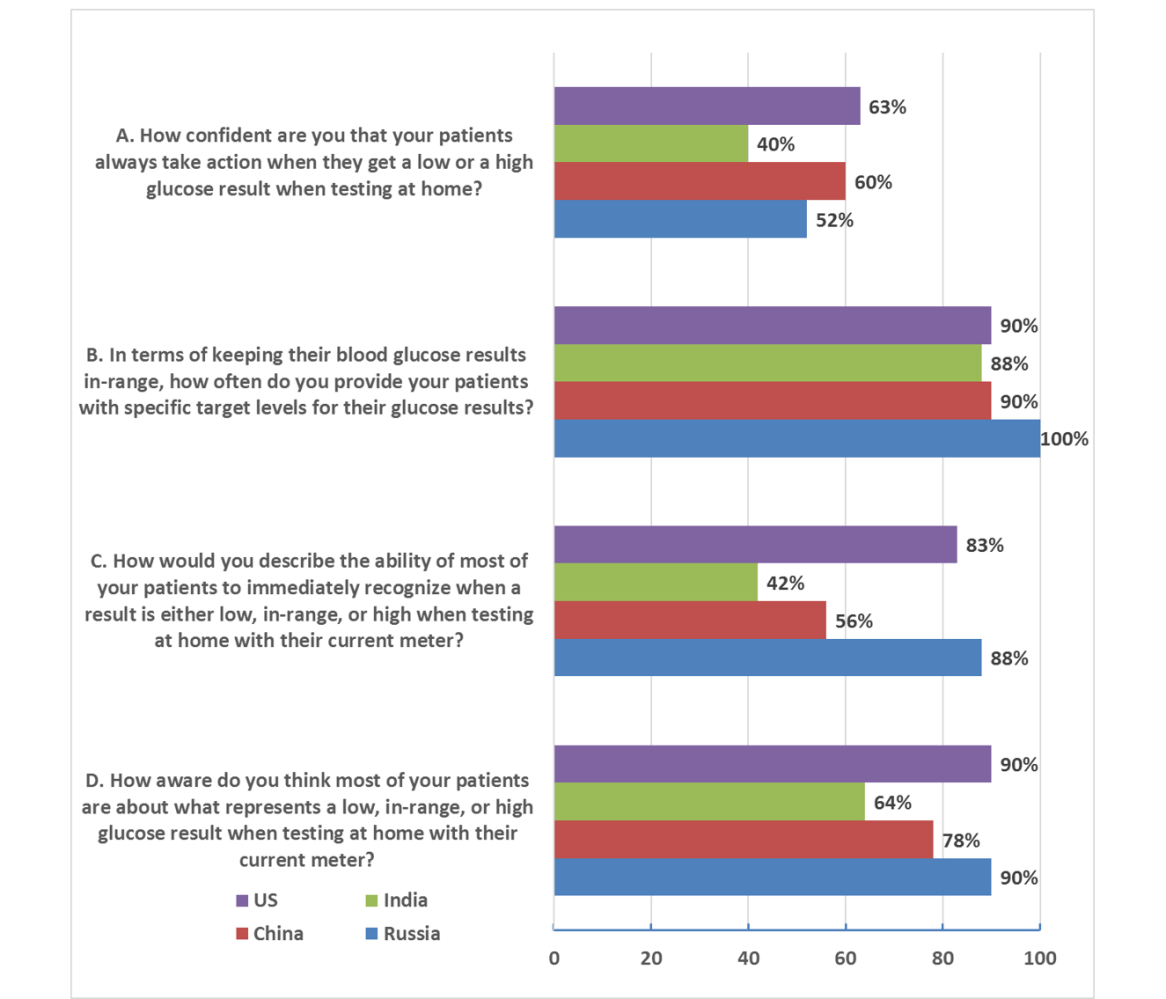
Response to prestudy clinical practice questions from 180 health care professionals (50 each from Russia, India, and China, and 30 from the United States). Responses are the top two positive responses (1 or 2) on a five-point scale for each question corresponding to (A) very confident or confident, (B) every time or most times, (C) very easy or easy, and (D) very aware or aware.

Of the HCPs in the United States and Russia, 90% (27/30 and 45/50, respectively) responded that their patients were either aware or very aware about what represents a low, in-range, or high glucose result when testing at home with their current meter compared to only 78% (39/50) in China and 64% (32/50) in India. A total of 83% (25/30) of HCPs in the United States and 88% (44/50) in Russia responded that most of their patients could immediately recognize when results were low, in range, or high when testing at home with their current meter compared to only 56% (28/50) or 42% (21/50) in China and India, respectively. Regardless of country, HCPs had similar responses when asked how often they personally provided their patients with specific target levels for their glucose results with 90% (27/30) of American, 100% (50/50) of Russian, 90% (45/50) of Chinese, and 88% (44/50) of Indian HCPs responding they provided this information most or every time they met. Furthermore, HCPs across all countries had low confidence that their patients took action when they got low or high glucose results at home, with only 63% (19/30) of American, 52% (26/50) of Russian, 60% (30/50) of Chinese, and 40% (20/50) of Indian HCPs having confidence their patients took action (Figure 3).

### Health Care Professionals’ Feedback During Online Interaction With the Meter

During the interactive online meter experience, 92% (46/50) of Russian, 90% (45/50) of Indian, 88% (44/50) of Chinese, and 63% (19/30) of American HCPs agreed that the easy-to-understand ColorSure technology could support patients’ ability to know when to act on their blood glucose results. In addition, 92% (46/50) of Russian, 90% (45/50) of Indian, 88% (44/50) of Chinese, and 63% (19/30) of American HCPs agreed a meter with color could help their patients feel more confident about managing their diabetes compared to receiving number results alone (Table 2). In all countries, HCPs often do not have ample time to teach patients about new technology. Therefore, it was valuable to 92% (46/50) of Russian, 86% (43/50) of Indian, 92% (46/50) of Chinese, and 67% (20/30) of American HCPs that this meter was so simple that the majority of their patients could start using it without additional training. Additionally, 96% (48/50) of Russian, 86% (43/50) of Indian, 86% (43/50) of Chinese, and 67% (20/30) of American HCPs agreed this meter could be used right out of the box without any additional instructions from them. Simple paper-based reminder tools to assist individual patients on how to react to different blood glucose results can support positive decision making. This meter comes with a paper reference card that allows HCPs to include personalized information on how individual patients should interpret or act on different levels of glucose results. All (100%, 50/50) of Russian, 84% (42/50) of Indian, 90% (45/50) of Chinese, and 73% (22/30) of American HCPs agreed such recommendations from them written on the reference card guide could help their patients know what to do next. Furthermore, 94% (47/50) of Russian, 82% (41/ 50) of Indian, 94% (47/50) of Chinese, and 80% (24/ 30) of American HCPs responded that recommendations from them in this paper guide could help their patients make the right decisions about their blood glucose results. In terms of overall benefits, 90% (45/50) of Russian, 86% (43/50) of Indian, 88% (44/50) of Chinese, and 60% (18/30) of American HCPs agreed that the meter itself provides patients with the added security of understanding their blood glucose numbers and provides reassurance about managing their diabetes.

**Table 1:** Health care professionals’ status and clinical practice information.

Health care professional information		Russia (n=50)	India (n=50)	China (n=50)	United States (n=30)	Total (N=180)
**Profession, n (%)**						
	Endocrinologist	30 (60)	30 (60)	30 (60)	15 (50)	105 (58)
	Primary care physician	8 (16)	8 (16)	8 (16)	10 (33)	34 (19)
	Diabetes educator	7 (14)	7 (14)	6 (12)	5 (17)	25 (14)
	Pharmacist^a^	5 (10)	5 (10)	6 (12)	—	16 (9)
**Gender (male), n (%)**						
	Endocrinologist	2 (7)	22 (73)	12 (40)	9 (60)	45 (43)
	Primary care physician	4 (50)	4 (50)	6 (75)	7 (70)	21 (62)
	Diabetes educator	0 (0)	3 (43)	1 (17)	0 (0)	4 (16)
	Pharmacist^a^	2 (40)	5 (100)	4 (67)	—	11 (69)
**Age (years), mean (SD** ^b^ **)**						
	Endocrinologist	41 (10)	42 (4)	39 (6)	47 (11)	41 (8)
	Primary care physician	38 (7)	43 (4)	36 (8)	47 (9)	41 (8)
	Diabetes educator	33 (7)	37 (5)	42 (12)	40 (9)	37 (9)
	Pharmacist^a^	34 (8)	39 (3)	35 (3)	—	36 (5)
**Years in current role, mean (SD)**						
	Endocrinologist	14 (9)	12 (3)	14 (7)	16 (8)	13 (7)
	Primary care physician	12 (7)	14 (4)	13 (7)	17 (8)	14 (7)
	Diabetes educator	11 (7)	10 (2)	18 (6)	14 (10)	13 (7)
	Pharmacist^a^	11 (9)	10 (2)	7 (3)	—	10 (5)
**Patients with diabetes, T1D%/T2D%** ^c^						
	Endocrinologist	18/82	30/70	8/92	28/72	20/80
	Primary care physician	21/79	32/68	6/94	14/86	18/82
	Diabetes educator	46/54	27/73	18/82	34/66	32/69
	Pharmacist^a^	34/66	21/79	16/84	—	23/77
**Patient therapy, %** ^c^						
	Medications and insulin	25	33	35	33	31
	Insulin only	29	23	15	25	23
	Medications only	40	28	41	35	36
	Not on any medications/insulin	5	14	7	6	9
	Other (eg, lifestyle)	1	2	2	1	1

^a^ Pharmacists were not recruited as part of the US cohort of HCPs.

^b^SD: standard deviation

^c^Percentages shown are estimates given by the HCPs.

**Table 2:** Health care professionals’ responses to 25 survey statements about the OneTouch Select Plus Simple meter. Results shown are percentage favorable responses (“strongly agree” or “agree”) on a five-point scale where 1=strongly agree, 2=agree, 3=neither agree nor disagree, 4=disagree, and 5=strongly disagree. All percentage favorable responses are statistically significant (ie, lower bound of 95% confidence limits >50%)

Survey statements		Favorable response, n (%)				
		Russia (n=50)	India (n=50)	China (n=50)	United States (n=30)	Total (N=180)
With security from understanding their blood glucose results, patients will feel confident in managing their diabetes		45 (90)	41 (82)	39 (78)	18 (60)	142 (79)
ColorSure technology shows patients when they are in range (green) and gives positive feedback which may help to keep them on track		43 (86)	37 (74)	46 (92)	21 (70)	148 (82)
Patients will feel reassured using this meter because of the ColorSure technology, audio signals, and it is so simple and easy to use right out of the box		49 (98)	41 (82)	42 (84)	22 (73)	155 (86)
Recommendations from me, written in the Reference Card Guide could help my patients know what to do next		50 (100)	42 (84)	45 (90)	22 (73)	158 (88)
This meter with ColorSure technology helps patients feel more confident about managing their diabetes than numbers alone		47 (94)	40 (80)	44 (88)	25 (83)	157 (87)
With this meter, patients can feel secure because they can see and hear when they may need to act		48 (96)	41 (82)	47 (94)	22 (73)	158 (88)
With ColorSure technology to help them understand their numbers, a beep to tell them when they may need to take action, and reference card, patients can feel reassured		47 (94)	42 (84)	44 (88)	18 (60)	151 (84)
This meter helps tell patients when they may need to act and when they may be good to go		42 (84)	45 (90)	47 (94)	19 (63)	153 (85)
The small and slim design with large, easy-to-read numbers will help this meter fit into my patient’s life		44 (88)	41 (82)	45 (90)	18 (60)	148 (82)
Easy-to-understand ColorSure technology could support patients to know when to act on their blood glucose results		46 (92)	45 (90)	44 (88)	19 (63)	155 (86)
Patients would feel secure when using this meter because it has ColorSure technology and audio signals		41 (82)	42 (84)	42 (84)	18 (60)	142 (79)
With this meter, patients can feel reassured because they can see and hear if they may need to act		43 (86)	40 (80)	44 (88)	18 (60)	146 (81)
This meter provides patients with the added security of understanding their blood glucose numbers and reassurance about managing their diabetes		45 (90)	43 (86)	44 (88)	18 (60)	149 (83)
The meter is so straight forward, it could be used right out of the box without any additional instructions from me		48 (96)	43 (86)	43 (86)	20 (67)	155 (86)
This meter will help patients to feel confident about their blood glucose result/ about managing their diabetes, they just insert a test strip to get started		47 (94)	42 (84)	46 (92)	19 (63)	155 (86)
Easy-to-understand ColorSure technology helps patients to know when they may need to act on their blood glucose results		44 (88)	41 (82)	45 (90)	20 (67)	149 (83)
Patients will feel a sense of security using this meter because of the ColorSure technology, audio signals, and it is so simple and easy to use right out of the box		44 (88)	39 (78)	48 (96)	21 (70)	151 (84)
The audio signal makes it clear when results are high or low so that patients can consider when to take action		47 (94)	44 (88)	46 (92)	25 (83)	162 (90)
Recommendations from me written in the reference card guide could help my patients make the right decisions about their blood glucose results		47 (94)	41 (82)	47 (94)	24 (80)	158 (88)
This meter is so simple, the majority of my patients could start using it without additional training		46 (92)	43 (86)	46 (92)	20 (67)	155 (86)
Using a meter with ColorSure technology helps patients feel more secure about managing their blood sugar levels than a meter without ColorSure technology		48 (96)	43 (86)	47 (94)	18 (60)	157 (87)
This meter provides patients with the added reassurance of understanding their blood glucose numbers and confidence about managing their diabetes		47 (94)	42 (84)	44 (88)	18 (60)	151 (84)
This meter brings clear understanding of results for my patients with sight and sound		47 (94)	40 (80)	47 (94)	18 (60)	151 (84)
With the reassurance from understanding their blood glucose results, patients will feel confident in managing their diabetes		45 (90)	47 (94)	45 (90)	19 (63)	157 (87)
The meter is a simple first step to understanding blood sugar results		46 (92)	39 (78)	46 (92)	20 (67)	151 (84)

**Figure 4:**
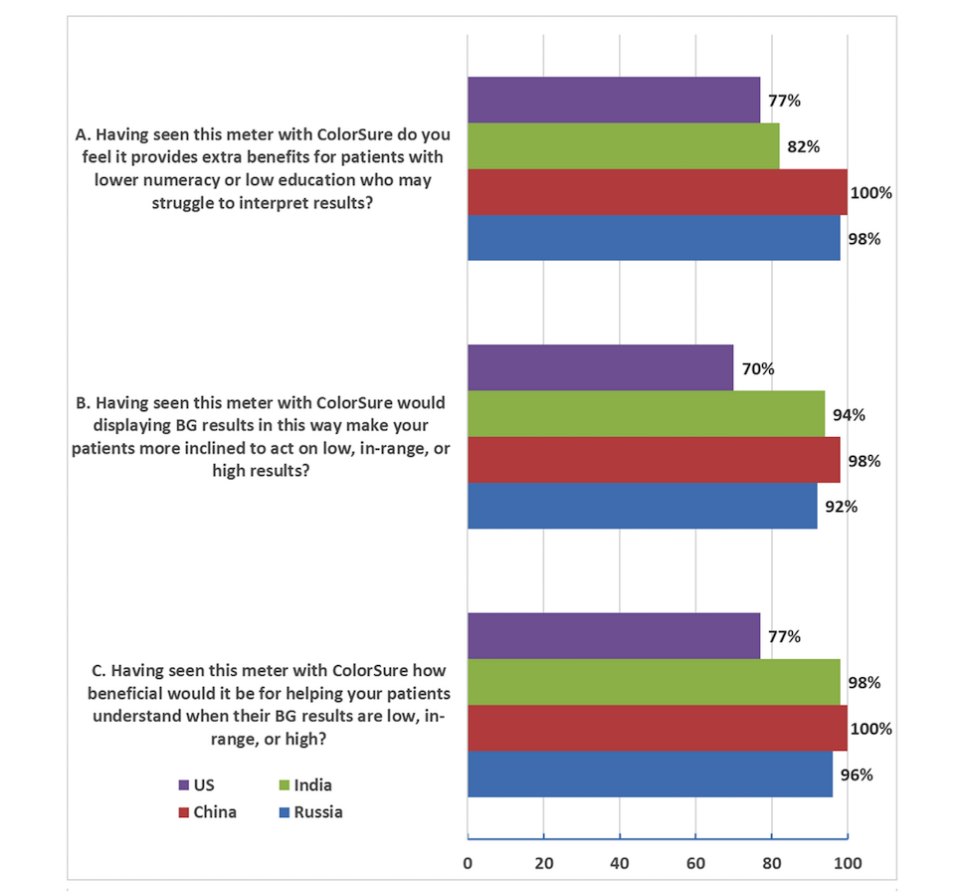
Response to clinical practice questions from 180 health care professionals after online experiences with a glucose meter with ColorSure (50 each from Russia, India, and China, and 30 from the United States). Responses are the top two positive responses (1 or 2) on a five-point scale for each question corresponding to (A) very beneficial or beneficial and (B and C) strongly agree or agree.

### Health Care Professionals’ Clinical Practice Outlook for Patients Based on Meter Experience

After experiencing the meter, 100% (50/50) of Chinese, 98% (49/50) of Indian, 96% (48/50) of Russian, and 77% (23/30) of American HCPs responded that their patients would find it beneficial to help them understand when their glucose results were low, in range, or high (Figure 4). In terms of taking action, 98% (49/50) of Chinese, 94% (47/50) of Indian, 92% (46/50) of Russian, and 70% (21/30) of American HCPs responded that displaying results with a color range indicator would make their patients more inclined to act on low or high glucose results. Finally, with respect to patients with low numeracy or low education, 100% (50/50) of Chinese, 98% (49/50) of Russian, 82% (41/50) of Indian, and 77% (23/30) of American HCPs agreed that a meter with a color range indicator could provide extra benefits for those patients who may struggle to interpret glucose results.

## Discussion

The methodology of this online study represents a novel, interactive approach to rapidly obtaining clinical insights from a diverse group of HCPs across multiple countries. The data provide evidence that HCPs from four countries had high overall satisfaction with this new glucose meter and specifically confirmed that using color-coded information to assist patients with interpreting their blood glucose information is a strategy that resonates universally with HCPs working in a variety of different health care environments.

The findings also highlight similarities and differences among HCPs from these countries regarding their patients’ basic comprehension of diabetes self-management, particularly glucose monitoring. The HCPs in the United States and Russia were more positive regarding their patients’ awareness of what constituted a low or high result than those in either India or China. Similarly, there was a higher confidence expressed by HCPs in the United States and Russia compared to those in India or China concerning the ability of patients to immediately recognize low, in-range, or high blood glucose results. The factors influencing regional differences are likely complex, but may relate to access of patients to health care, self-monitoring of blood glucose training, or issues relating to education, health literacy, or socioeconomic status. These issues are often barriers to health outcomes in different geographic regions [2-6,13-15]. Interestingly, regardless of country, HCPs provided similar positive responses with respect to their own efforts to consistently provide their patients with glycemic targets during routine visits suggesting that HCPs across these countries believe they are doing well with respect to goal setting and delivery of care. However, HCPs in India and China gave appreciably lower scores regarding their patients’ ability to recognize low or high blood glucose results than those in the United States, which may reflect underlying shortcomings in self-care behaviors, educational level, or numeracy in these countries, particularly in rural areas.

Another common finding related to patient behavior was that HCPs had limited confidence that their patients take action at home in response to low or high results. Regardless of country, HCPs believe that their patients display a reluctance to act on self-monitoring data and this remains a barrier to progress. The glucose meter that HCPs experienced in our study was designed to overcome such barriers to patient understanding by using a simple color range indicator to improve patient interpretation and awareness of glucose results [12].

One of the goals of this study was to discover which aspects of this color-enhanced glucose meter resonated most with HCPs and would be most beneficial for their patients. The HCPs agreed that the color range indicator could help patients feel more confident about managing their diabetes than simply numbers alone and could support patients knowing when to act on results. The HCPs felt their patients may not know whether a result is low, in range, or high; therefore, immediate reinforcement using color coding could help patients recognize the significance of their blood glucose results. Furthermore, over time patients may become more familiar with how color-coded glucose results relate to glycemic risk and may become better able to tell their HCP when they experienced low or high results and what actions or behaviors coincided with these results.

Clinicians understandably focus predominantly on low or high glucose fluctuations for reasons of patient safety. But highlighting in-range (green) results could stimulate patient motivation and reinforce beneficial behaviors. This resonated with HCPs in that they agreed that such positive feedback might help patients feel more secure and could be more helpful in managing their glucose than a meter without color. Patients are receptive to praise and encouragement; however, this does not always occur during office visits. A US study found only 77% of noninsulin and 83% of insulin-using patients regularly received encouragement to check blood glucose, with only 58% and 63% regularly receiving any congratulations from their HCP for checking blood glucose [16]. Achieving blood glucose results within the green zone might provide recognition for patients of positive behavior between relatively infrequent HCP visits. The HCPs agreed that personal recommendations from them, hand-written in the OneTouch Select Plus Simple reference guide, could help patients know what to do next or to make the right decisions between office visits.

Even within health care systems in developed countries, encounters with HCPs are of short duration. An analysis of 46,250 adult visits to primary care physicians in the United States between 1997 and 2005 calculated a mean visit duration of 18.9 minutes for a general examination, extended by only 4.2 minutes on average for patients with diabetes [17]. Furthermore, an International Diabetes Foundation report cautioned that the burden on endocrinologists employed in large Russian cities will be inappropriately heavy (up to 1500 patients for each endocrinologist), which would reduce the time that each physician could allow for one patient to approximately 10 minutes [18]. An additional issue was highlighted in a study in Russia, which found 63% of people with diabetes had not participated in any diabetes education compounding the effects of lack of access to a HCP [19]. It is likely that access to and time with an HCP is probably diminished even further for patients in rural areas and/or developing countries such as India or China, although reliable data on provision of care in these regions is scarce. These circumstances may partly explain why HCPs were so positive regarding the simple paper reference card supplied with the OneTouch Select Plus Simple meter containing HCP reminders for patients about what to do in response to low, in-range, or high glucose results. This could become a valuable educational tool for the HCP to reassure patients between relatively infrequent and short face-to-face consultations.

After participation in the online meter experience, all 180 HCPs were asked to consider how color-coded information might benefit their patients. There was universal appreciation that color could help patients better understand when results were low, in range, or high, and agreement that associating results with color might make patients more inclined to act on results. It is worth noting that HCP responses in Russia, India, and China to both closing questions were consistently between 92% and 100% (46/50-50/50), whereas HCPs in the United States gave positive, but appreciably lower, responses at 70% to 77% (21/30-23/30). The lower responses from American HCPs might be explained by a higher confidence in their ability to deliver care given greater access to resources, new technologies, and educational support to patients. Therefore, they may feel the benefits of color coding glucose information is less a priority in their own clinical practice compared to the circumstances faced by HCPs in other countries. A similar picture emerges with respect to the benefit of color for patients with low education and/or numeracy skills. American HCPs were less positive than the three other regions regarding these benefits. It is clear from the UNESCO report on education [11] and data specific to diabetes numeracy [8-10] that health inequality is an issue not only for those living in rural or developing regions with poor access to health care advice or technologies, but also for those who have access but simply lack the ability to interpret the results shown on these technologies.

The study recruited a lower number of American HCPs because the meter is not planned to be available in the United States. The inclusion of HCPs from the United States was intended predominantly for comparative purposes as an example of HCP attitudes and perceptions in a country with more consistent care provision.

In conclusion, this multicountry online study provides evidence that HCPs had high overall satisfaction with the OneTouch Select Plus glucose meter, which uses color-coded information to assist patients with interpreting blood glucose results. This may be especially helpful in patient populations with low numeracy or literacy and limited access to health care and direct interaction with HCPs.

## Abbreviations

HCP: health care professional
T1D: type 1 diabetes
T2D: type 2 diabetes
